# Protocol for imaging nuclear pore complexes on isolated nuclei of zebrafish early embryos by field emission scanning electron microscopy

**DOI:** 10.1016/j.xpro.2023.102341

**Published:** 2023-06-13

**Authors:** Weimin Shen, Anming Meng

**Affiliations:** 1 Laboratory of Molecular Developmental Biology, State Key Laboratory of Membrane Biology, Tsinghua-Peking Center for Life Sciences, School of Life Sciences, Tsinghua University, Beijing 100084, China; 2 Laboratory of Stem Cell Regulation, Guangzhou Laboratory, Guangzhou 510320, China

**Keywords:** Cell Biology, Cell separation/fractionation, Developmental biology

## Abstract

Here, we present a protocol to observe the three-dimensional surface of nuclear pore complexes (NPCs) of vertebrate early embryos by field emission scanning electron microscopy (FESEM). We describe steps from zebrafish early embryo collection and nuclei exposure to FESEM sample preparation and final NPC state analysis. This approach provides an easy way to observe surface morphology of NPCs from the cytoplasmic side. Alternatively, additional purification steps after nuclei exposure supply intact nuclei for further mass spectrometry analysis or other utilization.

For complete details on the use and execution of this protocol, please refer to Shen et al.^1^

## Highlights

• Exposure and collection of intact nuclei from zebrafish early embryos
• High-resolution imaging of nuclear pore complexes by scanning electron microscopy
• Step-by-step instructions to analyze NPC complexity by size and morphology
• Additional isolation of pure and intact nuclei for mass spectrometry

## Abstract (editor highlights)

Publisher’s note: Undertaking any experimental protocol requires adherence to local institutional guidelines for laboratory safety and ethics.

## Graphical abstract

**Figure:**
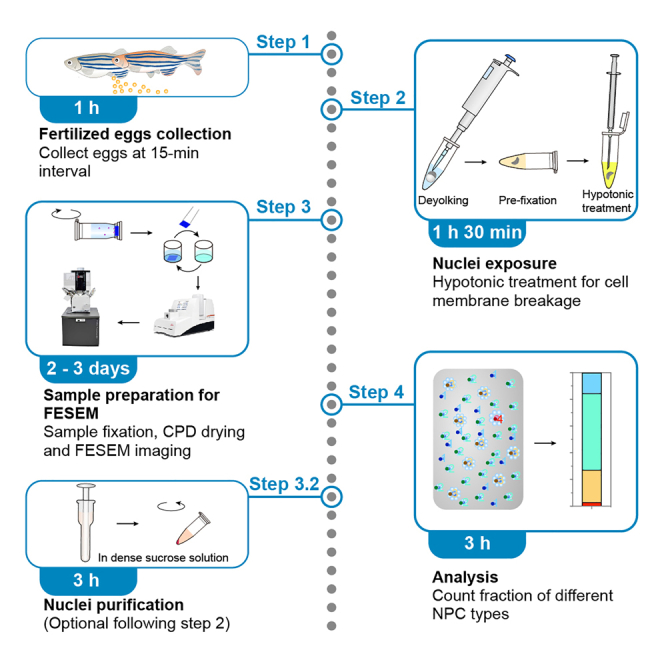

## Before you begin

Nuclear pore complexes (NPCs), channels for nucleocytoplasmic transport embedded in the nuclear envelope (NE), have a high degree of heterogeneity within a nucleus or among different nuclei and are important for cell identity.^2^^,^^3^^,^^4^ Surface images of NPCs obtained by FESEM are useful for analyzing NPC size and structural morphology. Previously described approaches employ FESEM imaging on nuclei isolated from cultured cells, large *Xenopus* oocytes or *in vitro* assembled nuclei.^5^^,^^6^ This protocol describes the optimized steps for exposing embryonic nuclei after cell membrane rupture by hypotonic treatment without use of detergents. These nuclei are derived from zebrafish early embryos around mid-blastula transition (MBT, about 512-cell to 1k-cell stage). The exposed nuclei are then collected for FESEM analysis. Alternatively, intact nuclei can be further purified by high-speed centrifugation using an optimized sucrose method^7^ for LC-MS/MS analysis. In our experience, the nuclei of vertebrate early embryos before MBT are much more fragile than those after MBT or from somatic cells. We also used this protocol to efficiently isolate nuclei from zebrafish embryos at the gastrula stage, indicating its fitness for somatic or cultured cells.

### Institutional permissions

All zebrafish experiments were conducted under a protocol approved by the IACUC (Institutional Animal Care and Use Committee) of Tsinghua University.

### Zebrafish breeding for embryo collection

**Timing: 2 h (the day before embryos are collected)**

1. Assemble breeding tanks the night before and fill tanks with system water to an appropriate depth so that fish can swim freely.

***Note:*** The breeding tank consists of a bottom tank and a flexible, nested inner tank. The inner tank has a lattice bottom, allowing embryos to fall into the bottom tank for ease of embryo collection and also preventing adult fish from eating eggs.

**Figure 1:**
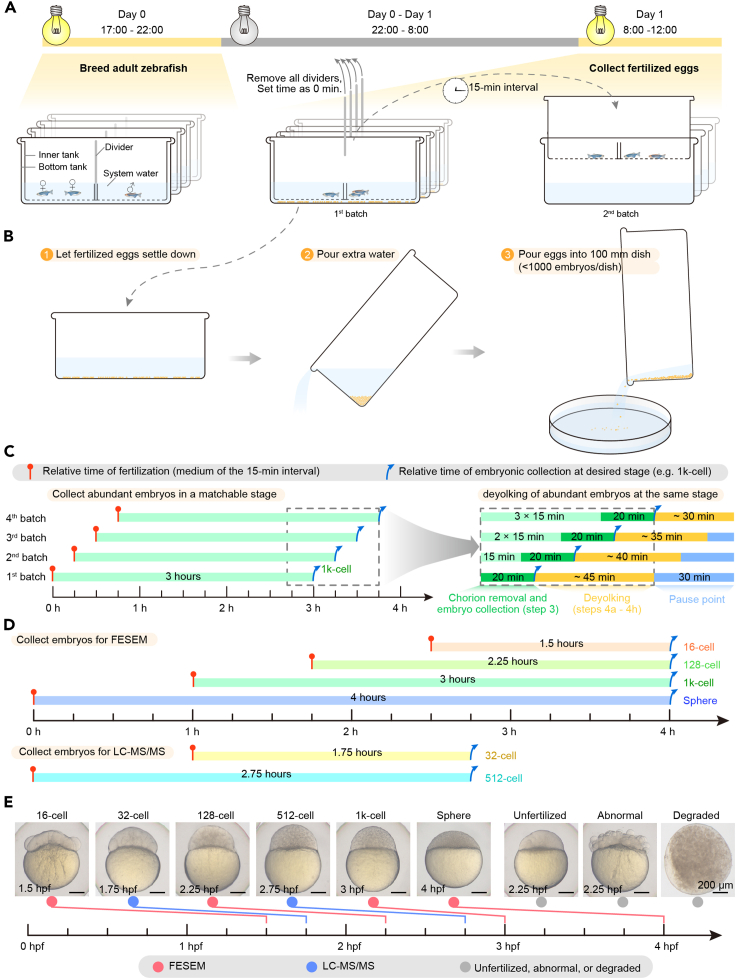
Schematic illustration of adult zebrafish breeding, fertilized egg and desired embryo collection (A) Schematic for zebrafish breeding the night before and spawning control the following morning. (B) Schematic of fertilized egg collection. (C) Schematic of schedule for collecting and deyolking embryos of the same stage from different batches. Fertilized egg collections were separated by 15-min interval (left), then the relevant embryos were collected at 15-min interval for deyolking (right). The axis at the bottom-left indicates the relative hours. Dotted grey box in the left is depicted in detail in the right. Time for deyolking, orange bars in the right panel, decreased among different batches due to reduced embryonic number (right). Red lollipops, relative time of fertilization; blue arrow, relative time of embryonic collection at the desired stage. (D) Schematic for isolating nuclei from embryos at different stages on a single day. (E) Representative images of wild-type embryos used in this protocol, and unfertilized, abnormal, or degraded embryos. hpf, hours post fertilization. Scale bar, 200 μm.

***Optional:*** Insert a divider in the central region of the inner tank to separate male and female fish (Figure 1A).

2. Feed the fish, then transfer a single male and one or two female fish into opposite sides of an assembled tank (Figure 1A).
  a. Zebrafish are photoperiodic and are maintained in a 14-h light / 10-h dark cycle at about 28.5°C.
  b. Place fish into breeding tanks between 17:00 and 22:00 before light is turned off.
  c. Generally, breeding fish at 6–12 months old would produce the maximal number of embryos.
  d. Female fish can be re-used 7–10 days after one spawning.
  e. Number of breeding pairs is dependent on experimental purpose.

***Note:*** Generally, one pair of fish produces 200–300 embryos. For FESEM, the suggested number of embryos are about 1000, 500, 150 and 50 for 16-cell, 128-cell, 1k-cell and sphere stages, respectively. For liquid chromatography-tandem MS (LC-MS/MS) analysis, about 30,000 embryos at the 32-cell stage and 1,900 embryos at the 512-cell stage are required for each replicate, respectively, as performed in our previous study.^1^

### Prepare silicon chips

**Timing: 50 min**

3. Mark individual silicon chips (each 5 × 5 mm^2^) by engraving symbols on the back surface with a diamond pen for sample tracking.
4. Wash the chips with ultrapure water for 3 times.
5. Pipette 20 μL 0.2 mg/mL poly-L-lysine onto the front surface of the chips. Spread the solution over the whole surface of each chip, and incubate for 30 min at room temperature (typically 20°C–25°C in this protocol).
6. Wash the chips with ultrapure water for 3 times. Air dry.

### Prepare improvised centrifugation holders

**Timing: 10 min**

**Figure 2:**
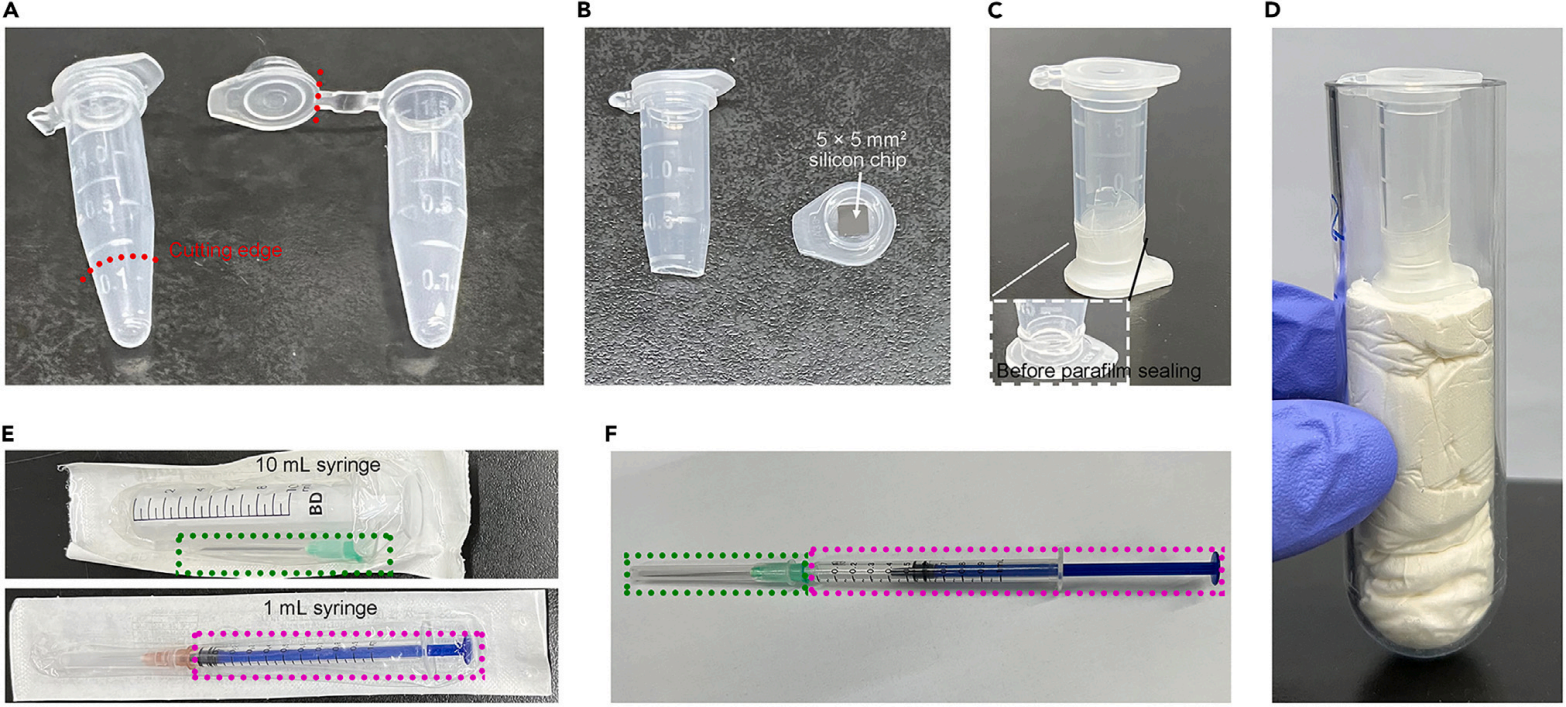
Assembly of an improvised centrifugation holder or 21-G needle connected syringe (A–D) Assembly of an improvised centrifugation holder. (A) Cut the bottom of a 1.5-mL EP tube below the 0.25 mL marker, and the cap from another tube. (B) Put the silicon chip with poly-L-lysine coated side facing upwards inside the cap. (C) Place the bottom cut tube inside the cap and ensure that it fits snugly to prevent leakage. Wrap parafilm around the connecting region. (D) This device is spun in a swing-bucket rotor by placing it into a 50-mL tube stuffed with tissue papers. (E and F) Assembly of a 21-G needle device. The 21-G needle from a 10-mL syringe is connected to a 1-mL syringe cylinder for cell membrane rupture.

This step describes the preparation of an improvised centrifugation holder for each individual silicon chip to thoroughly spread nuclei over the surface of poly-L-lysine coated silicon chip (Figures 2A–2C), and see also in Shaulov et al.^6^

7. Cut the bottom of a 1.5-mL Eppendorf (EP) tube below the 250-μL mark, and cut the cap from another EP tube.
8. Put the silicon chip with the poly-L-lysine coated surface facing upwards inside the separated cap.
9. Assemble the holder by inserting the bottom-cut tube into the separated cap containing silicon chip.
10. Wrap the connecting region of the holder with parafilm to prevent leakage.
11. Fill the holder with 900 μL PBS + 10% glycerol before the addition of treated cells.

***Note:*** Fully seal the device.

## Key resources table

**Table:** 

REAGENT or RESOURCE	SOURCE	IDENTIFIER
**Chemicals, peptides, and recombinant proteins**		

Sodium chloride (NaCl)	Beijing Tong Guang	Cat # 11148
Potassium chloride (KCl)	Beijing Tong Guang	Cat # 11295
Calcium chloride (CaCl_2_)	Vetec	Cat #V900266
Sodium bicarbonate (NaHCO_3_)	Beijing Tong Guang	Cat # 11162
Tris	Amresco	Cat # 0497
HEPES	Sigma	Cat #H3375
Magnesium chloride hexahydrate (MgCl_2_·6H_2_O)	Beijing Chemical Industry	Cat #S1718
Sucrose	Amresco	Cat # 0335
Glycerol	Sigma	Cat # 49767
DPBS	Gibco	Cat # 21600044
Pronase	Sigma	Cat #P5147
Protease inhibitor cocktail	Roche	Cat # 04693132001
Poly-L-lysine hydrobromide	Sigma	Cat #P1524
8% Paraformaldehyde (PFA) solution	EMS	Cat # 157-8
8% Glutaraldehyde (GA) solution	EMS	Cat # 16020
Sodium dihydrogen phosphate (NaH_2_PO_4_)	Sigma	Cat #V900328
Disodium hydrogen phosphate (Na_2_HPO_4_)	Sigma	Cat #V900061
OsO_4_	EMCN	Cat # GP18456
Sodium cacodylate	SPI-CHEM	Cat # GA18139
DAPI	Thermo Fisher	Cat #D1306
Wheat germ agglutinin Alexa Fluor 647	Thermo Fisher	Cat #W32466

**Experimental models: Organisms/strains**		

Zebrafish: wild-type AB strain, 6 to 12-month-old	N/A	N/A

**Software and algorithms**		

Fiji	Schindelin et al.^8^	https://imagej.net/Fiji
Prism 8	GraphPad	https://www.graphpad.com/scientificsoftware/prism/

**Other**		

1 mL syringe	Zhiyu	Cat # HC0101
10 mL syringe (21-G needle)	BD	Cat # 301947
40-μm Cell strainer	BD Falcon	Cat # 352340
High precision tweezers	WPI	Cat # 14099
1.5 mL, polypropylene tube with snap-on cap	Beckman	Cat # 357448
Silicon chips, 5 × 5 mm^2^	EMCN	Cat # BZS0505
Eppendorf centrifuge 5430 R (with a fixed-angle rotor)	Eppendorf	N/A
Eppendorf centrifuge 5810 R (with a swing-bucket rotor)	Eppendorf	N/A
Confocal dish	In Vitro Scientific	Cat #D35-20-1.5-N
Critical Point Dryer Leica EM CPD300	Leica Microsystems	N/A
FEI Helios NanoLab G3 UC	FEI Company	N/A
Optima MAX-XP tabletop ultracentrifuge	Beckman	N/A

## Materials and equipment

***Alternatives:*** This protocol uses many chemical reagents for buffer preparation. All of these chemicals are alternatively supplied by other suppliers. Most of materials and equipment are also alternatively obtained from other suppliers. However, the ultracentrifugation tube must be the one designed for associated ultracentrifuge.

**Table:** Holtfreter’s solution

Reagent	Final concentration	Amount
NaCl	3.5 g/L	3.5 g
KCl	0.05 g/L	0.05 g
CaCl_2_	0.1 g/L	0.1 g
NaHCO_3_	0.025 g/L	0.025 g
ddH_2_O	N/A	1 L
**Total**	**N/A**	**1 L**

Adjust pH to 7.0–7.4 with about 7 μL 6 M HCl. Store at room temperature for up to 2 days.

**Table:** Pronase stock solution (30×)

Reagent	Final concentration	Amount
Tris (1 M stock solution, pH7.0)	10 mM	1 mL
NaCl (5M stock solution)	10 mM	200 μL
Pronase (Sigma, P5147)	30 mg/mL	3 g
ddH_2_O	N/A	98.8 mL
**total**	**N/A**	**100 mL**

Incubate the above mixture in water bath at 37°C for 1 h for complete dissolution of pronase powder. Divide this stock solution into 1 mL aliquots, and store at −20°C.

**Table:** Ringer’s solution (Ca^2+^ free)

Reagent	Final concentration	Amount
NaCl (5M stock solution)	116 mM	4.64 mL
KCl (2M stock solution)	2.9 mM	290 μL
HEPES (0.1M stock solution)	5 mM	10 mL
ddH_2_O	N/A	185.07 mL
**Total**	**N/A**	**200 mL**

Adjust pH to 7.2. Filter through a 0.22-μm filter. Store at room temperature for up to 1 month. All the solutions used for nuclei isolation or FESEM sample preparation should be filtered through a 0.22-μm filter.

**Table:** Hypotonic buffer

Reagent	Final concentration	Amount
Tris-HCl (1 M stock solution, pH 7.5)	15 mM	750 μL
NaCl (5 M stock solution)	10 mM	100 μL
MgCl_2_ (1 M stock solution)	3 mM	150 μL
ddH_2_O	N/A	49 mL
**Total**	**N/A**	**50 mL**

Adjust pH to 7.4. Filter through a 0.22-μm filter. Pre-chill in the ice before use.

**CRITICAL:** This solution is best prepared on the day of use. Or divide the fresh prepared solution into 10 mL aliquots, store at −20°C, and avoid freeze / thaw cycles to prevent formation of aggregates or particles.

**Table:** Hypertonic sucrose buffer

Reagent	Final concentration	Amount
Sucrose	2.2 M	37.653 g
Tris-HCl (1 M stock solution, pH 7.5)	10 mM	500 μL
MgCl_2_ (1 M stock solution)	1 mM	50 μL
ddH_2_O	N/A	fill to 50 mL
**Total**	**N/A**	**50 mL**

Adjust pH to 7.2. Filter through a 0.22-μm filter. Store at 4°C for up to 1 month.

**Table:** 2% paraformaldehyde (PFA) + 2% glutaraldehyde (GA) solution

Reagent	Final concentration	Amount
8% PFA solution	2%	250 μL
8% GA solution	2%	250 μL
PB (0.2 M, pH 7.4)	0.1 M	500 μL
**Total**	**N/A**	**1 mL**

Freshly prepare on the day of use and protect from light. Wear gloves, protective clothing and safety goggles when handling this solution in chemical hood.

• **PBS**: add 9.55 g DPBS powder (Gibco, 21600044) in 1 L ddH_2_O.

Filter through a 0.22-μm filter. Store at room temperature for up to 1 year.

• **PB**: mixture of 19 mL NaH_2_PO_4_ (0.2 M) and 81 mL Na_2_HPO_4_ (0.2 M).

The pH is about 7.4. Filter through a 0.22-μm filter. Store at room temperature for up to 1 year.

• **0.2 mg/mL Poly-L-lysine solution**: add 0.002 g Poly-L-lysine hydrobromide (Sigma, P1524) in 10 mL ddH_2_O

Filter through a 0.22-μm filter. Aliquot into 100 μL/tube. Store at −20°C for up to 6 months.

• **PBS + 10% glycerol**: add 5 mL glycerol in 45 mL PBS.

Filter through a 0.22-μm filter. Store at 4°C for up to 1 month.

• **0.2 M cacodylate buffer**: add 0.36 g sodium cacodylate in 10 mL ddH_2_O.

Adjust pH to 7.4. Filter through a 0.22-μm filter. Store at 4°C and protect from light for up to 1 year.

**CRITICAL:** Sodium cacodylate is toxic by ingestion, inhalation, and skin absorption. Wear gloves, protective clothing and safety goggles when handling this solution in chemical hood.

• **1 mg/mL wheat germ agglutinin Alexa Fluor 647 (WGA AF647) stock solution**: add 1 mg WGA AF647 (Thermo W32466) in 1 mL Nuclease-free water.

Aliquot into 2 μL/tube. Snap freeze in liquid nitrogen and store at −80°C for up to 1 year.

• **1 mg/mL DAPI solution**: add 10 mg DAPI (Sigma D9542) in 10 mL ddH_2_O.

Aliquot into 10 μL/tube. Store protected from light at −20°C for up to 1 year.

## Step-by-step method details

### Fertilized egg collection at 15-min interval

**Timing: 1 h**

This step describes egg collection to acquire a large number of embryos at desired stages for nuclei isolation.

1. Remove the divider for fish mating (Figure 1A):
  ***Note:*** Zebrafish initiate breeding after the onset of light. Mature oocytes of female fish would degrade without spawning after midnoon. Therefore, this step is generally done between 8 and 12 o’clock in the morning.
  a. Exchange water with fresh system water to remove excrement in the bottom tank.
  b. Prepare an equal number of alternative bottom tanks filled with fresh water for transferring inner tanks.
  c. Remove the dividers from all tanks quickly, and set a timer of 15 min for egg collection.
    ***Note:*** The duration of each cleavage before MBT is 15 min for zebrafish embryos at 28.5°C. Therefore, we set a 15-min interval for easy collection of abundant embryos at a matchable stage.

2. Collect eggs in batches at 15-min interval. Troubleshooting 1 and 2.
  a. Transfer all inner tanks to the alternative bottom tanks once the 15-min time is up (Figure 1A).
  b. Set the next 15-min timer again immediately.
  c. Collect fertilized eggs (Figure 1B).
    i. Pour most water and leave about 35 mL water to drown eggs, once fertilized eggs sink to the bottom of bottom tank .
    ii. Gently pour eggs into a 100-mm dish. Each dish can accommodate a maximum of 1000 eggs.
    iii. Mark every dish with spawning time, fish strain, approximate number of eggs, date, and owner’s name.
    iv. Exchange the water with Holtfreter’s solution, and incubate at 28.5°C.
  d. Repeat the above steps for 2 or 3 times once the next 15-min timer is up (Figure 1C, left).

***Note:*** Different batches of embryos can be pooled when they develop to a matchable developmental stage (Figure 1C, left). Fertilization time can be controlled by managing divider removal time to collect embryos at different developmental stages at the same time (Figure 1D).

### Nuclei exposure of zebrafish early embryos

**Timing: 1 h 30 min**

This step provides details on nuclei exposure from zebrafish early embryos at desired stages around MBT.

3. Collect zebrafish early embryos at desired stages with chorion detached:
  a. Monitor zebrafish early embryonic development under a microscope by referring to the classical developmental stage definition and developmental time at 28.5°C^9^ (Figure 1E).
    ***Note:*** Unfertilized eggs, abnormal or degraded embryos (Figure 1E) should be picked out in advance.
    ***Note:*** Several batches of fertilized eggs were collected with 15-min interval, so embryos from different batches were also collected with 15-min interval to achieve the same developmental stage. Therefore, the collection and deyolking processes of embryos from different batches are timely overlapped (steps 3a – 4h) (Figure 1C, right). Prepare the next batch when the previous one is in centrifugation, standing, or pause point.
    ***Note:*** About 20 min before the embryos reach a desired stage, move on to the next step.
  b. Prepare embryos for pronase treatment.
    i. Transfer embryos to a suitable dish, such as a 35-mm dish for about 2000 embryos.
    ii Aspirate surplus Holtfreter’s solution to a minimum level while the embryos are still submerged.
    iii. Add 30 mg/mL pronase stock solution into the Holtfreter’s solution to a final concentration of about 1 mg/mL.
      ***Note:*** Static electricity from plastic or scratches on the surface of recycled plastic dish may disrupt embryonic yolk. Therefore, glass dishes or plastic dishes coated with 3–4 mm thick 2% agarose, which is prepared in Holtfreter’s solution, are strongly recommended.
  c. Incubate at 28.5°C for about 7 min.
  d. Fill the dish with Holtfreter’s solution to dilute and wash out the reaction solution when chorion of about 1/3 embryos are broken.
    ***Note:*** Excessive digestion would be harmful to embryos and reduce the efficiency of nuclei isolation, so stop pronase digestion once partial chorions are detached. Shrunken chorions in the rest of embryos would be detached after being washed with Holtfreter’s solution.
  e. Wash embryos with Holtfreter’s solution for additional two or more times in order to remove detached chorion and residual pronase as much as possible.
    ***Note:*** Residual pronase would affect the efficiency of nuclei isolation.
  f. Transfer the embryos to a 1.5-mL EP tube on ice, after confirming that the majority of the embryos have reached the desired stage under a microscope.
    ***Note:*** Fertilized eggs were collected every 15 min, which is the length of one cleavage before MBT, so most embryos in one batch develop to a matchable stage at the same time. Furthermore, only embryos with formed nuclei, which are beyond mitosis, are suitable for nuclei isolation. Thus, it is better to quickly check by microscopy if most embryos stay at interphase, at which cells are just cleaved from the previous stage with obvious cell boundary, and then harvest embryos.
    **CRITICAL:** Transfer no more than 500 embryos into one 1.5-mL EP tube, otherwise efficient deyolking process may not be achieved. 500 chorion-removed embryos settle to a volume of about 0.1 mL. Generally, 1–8 EP tubes are set up for embryos at one desired stage in one batch.

4. Deyolk zebrafish early embryos:
  a. Aspirate the Holtfreter’s solution, and then wash embryos twice with ice-cold Ringer’s solution. Aspirate the Ringer’s solution.
  b. Add ice-cold Ringer’s solution with final 1× protease inhibitor cocktail (one cocktail tablet is dissolved in 1 mL ddH_2_O as 50× stock concentration) to each EP tube to a final volume of 1 mL.
  c. Add 67 μL 8% PFA to each EP tube to a final concentration of about 0.5% PFA. Troubleshooting 3.
    **CRITICAL:** This step significantly helps preserve early embryonic nuclei and enhances the efficiency of intact nuclei isolation. The thickness of the nuclear envelope in zebrafish early embryos before MBT is typically thinner than that after MBT.^1^ This may explain the reduced toughness of early embryonic nuclei.
  d. Pipette up and down as gently as possible with a 1-mL tip to disrupt yolk.
    ***Note:*** Pipetting should be as gentle as possible to prevent cell membrane or even nuclear envelope rupture. Initial deyolking is accomplished when no large yolk granules are visually observed, but it is acceptable for some tiny yolk granules to remain. It is better to transfer 10 μL of suspension into a 35-mm dish and examine blastomere cell integrity under a stereomicroscope.
    ***Note:*** For earlier stage embryos (e.g., 16-cell stage) with larger blastomeres, a 1-mL tip could be cut at the most distal tip (about 1 mm long) and polished by fire.
  e. Place the tube lying on bench top at room temperature for 5 min (starting the timer when PFA is added).
  f. Centrifuge at 500 × *g* for 5 min at room temperature. Aspirate the supernatant.
  g. Gently resuspend the pellet in 1 mL cold Ringer’s solution (containing protease inhibitor cocktail) with 1-mL tips.
  h. Pellet blastomeres by centrifugation at 500 × *g* for 5 min at 4°C. Aspirate the supernatant with minimal residual just covering the pellet.
    ***Optional:*** Wash blastomeres with ice-cold Ringer’s solution one more time if the yolk is not removed well. Blastomeres look white in the upper layer of the pellet, while the yolk looks pale yellow in the lower layer.
    **Pause point:** The pellet could stay on ice for up to about 30 min. This allows collection and deyolking of more embryos from other batches (Figure 1C, right), which were collected at 15-min interval in step 2.
  i. Gently resuspend the blastomere pellet in 200 μL ice-cold PBS once blastomeres from all batches are collected.
  j. Pool suspensions from up to 5 tubes into one EP tube, and centrifuge at 500 × *g* for 5 min at 4°C.
5. Hypotonic treatment of blastomeres for nuclei exposure:
  a. Aspirate PBS as much as possible.
    ***Note:*** Too much residual PBS would alter the concentration of hypotonic buffer and affect hypotonic treatment.
  b. Add 200 μL ice-cold hypotonic buffer into each tube.
  c. Gently resuspend the pellet by pipetting up and down with a 200-μL tip.
  d. Incubate on ice for 5 min.
  e. Gently pass blastomeres thrice through a 21-G needle connected to the hub of a 1-mL syringe (Figures 2E and 2F).
    ***Note:*** For other cell types, the length of incubation, the handling procedure of the needle, and the number of passes are required to be optimized empirically. A short incubation of 5 minutes and gentle handling of the needle for three times are sufficient for zebrafish early embryos to release nuclei, whereas a longer incubation and more harsh handling may be required for some tissue cells or culture cells.
  f. Pass the blastomere suspension through a 40-μm cell strainer.
    **CRITICAL:** This step removes large particles of undisrupted embryonic remnants such as residual yolk, which would otherwise compete for silicon chip attachment for FESEM or reduce the purity of nuclei for mass spectrometry.
  g. Centrifuge the filtrate at 1,500 × *g* for 5 min at 4°C.
    ***Note:*** The pellet contains a crude nuclear fraction likely contaminated with mitochondria and other membrane fractions.
    ***Note:*** The pellet is now ready for nuclei exposure onto the silicon chip for FESEM analysis. Alternatively, this crude nuclear pellet may be further purified for additional mass spectrometry analysis or other applications such as RNA-seq.

### Nuclei collection and sample preparation for FESEM

**Timing: 2–3 days**

This step is for nuclei preparation of FESEM analysis.

6. Load nuclei onto the silicon chip:
  a. Discard the supernatant.
  b. Gently resuspend the pellet in 100 μL of PBS + 10% glycerol.
  c. Transfer the cell suspension to 900 μL of PBS + 10% glycerol inside the improvised centrifugation holder (Figure 2C).
  d. Put the device inside a conventional 50-mL tube densely stuffed with facial tissues to a certain level (Figure 2D).
  e. Centrifuge at 800 × *g* for 10 min at 4°C in a swing-bucket rotor (e.g., Eppendorf 5810 R).

***Note:*** The nuclei and cell debris are spread onto the silicon chip at this step. The improvised centrifugation holder helps spread the nuclei and other residual cellular components.

**Figure 3:**
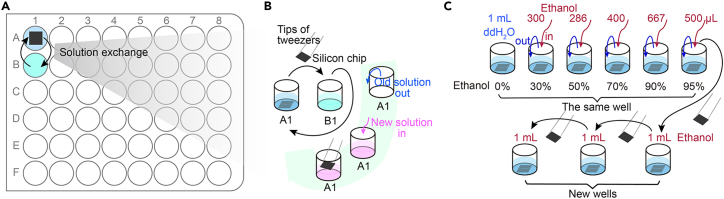
Schematic illustration of solution exchange for preparation of FESEM samples (A) Place silicon chips, with the nuclei attached side facing upwards, in the wells of a 48-well cell culture plate for FESEM sample preparation. Solution exchange is accomplished through the transfer of silicon chips between two wells. (B) Scheme of solution exchange involving the transfer of a silicon chip between two wells. For the first solution exchange, introduce solution into the second well (e.g., B1), and then transfer the silicon chip using a high precision tweezer to the second well (e.g., B1). For the second solution exchange, remove the solution from the first well (e.g., A1), replace it with new solution, and then transfer the chip again. Repeat the subsequent exchange in the same way. (C) Schematic of dehydration through a graded ethanol series. A graded ethanol series is achieved by removing some of the old solution and introducing the same amount of ethanol. Repeat for each step and final step in 100% ethanol is done by transferring chips between wells as shown in (B).

7. Pre-fixation of nuclei on the silicon chip (Figures 3A and 3B): Troubleshooting 4.
  a. Carefully transfer the silicon chip into a 48-well cell culture plate filled with 1 mL PBS for washing.
    ***Note:*** Use high precision tweezers to transfer the silicon chip. Keep track of the chip and make sure not to turn over the chip.
  b. Transfer the chip to another well in the plate filled with 2% PFA + 2% GA solution. Fix for 30 min at room temperature, or overnight (typically 8–16 h in this protocol) at 4°C under dark condition.
    ***Note:*** Avoid the chip being dried during all processes. To achieve this goal, we transfer the chip using high precision tweezers between two wells filled with solution in advance.

8. Post-fixation of nuclei on the silicon chip:
  a. Wash the chip thrice in 0.1 M cacodylate buffer for 2 min each.
    ***Note:*** Add an equal volume of ddH_2_O to 0.2 M cacodylate buffer for a final concentration of 0.1 M.
  b. Post-fix samples in 1% OsO_4_ in 0.1 M cacodylate buffer for 10 min at room temperature under dark condition.
    ***Note:*** Mix an equal volume of 2% OsO_4_ and 0.2 M cacodylate buffer freshly.
    ***Note:*** OsO_4_ is highly toxic. Store at −20°C and be protected from light anytime. Wear gloves, protective clothing, and safety goggles to handle this solution in a chemical hood at any step. The liquid waste should be wet-sealed by vegetable oil and treated according to institutional instruction.
  c. Transfer the silicon chip to a new 48-well cell culture plate filled with ddH_2_0 and wash for 2 min.
    ***Note:*** The ddH_2_O should be filtered through a 0.22-μm filter before use.
    ***Note:*** The used plate should be properly treated according to institutional instruction after OsO_4_ treatment.
  d. Repeat ddH_2_O wash for additional two times, 2 min each.
9. Dehydration and critical point drying (CPD):
  a. Dehydrate through a graded ethanol series: 30%, 50%, 70%, 90% and 95%, 3 min each, and twice repeat for each step. Finally, transfer the silicon chip to 100% ethanol, 6 × 3 min (Figure 3C).
    **Pause point:** Samples can be stored in 100% ethanol overnight.
  b. Transfer the silicon chip to a CPD apparatus (e.g., Leica EM CPD 300) filled with ethanol for drying.
    ***Note:*** Exchange with high purity liquid CO_2_ for 12 times to ensure the complete exchange of fluid. Finally, the CO_2_ is converted to gas under its critical point of specific temperature and pressure so that the specimens on the chip get dried.
    ***Note:*** The samples will be examined with FESEM under vacuum, and thus the fixed biological samples must be fully dried beforehand. However, if aqueous biological samples are directly air-dried, the surface of the specimens would be damaged due to surface tension.
    ***Note:*** The critical point is the intrinsic physical property of a liquid. It is the specific pressure and temperature that allow a liquid and its vapor to coexist. The critical point of water is 374 °C and 229 bar, but CO_2_’s critical point is 31°C and 74 bar, making it more compatible with biological samples. However, water and CO_2_ are not miscible, so ethanol is used as an intermediary fluid. The aqueous biological samples should be dehydrated first through a graded series of ethanol.
    ***Note:*** The dried samples should be processed to the next step of sputter coating as soon as possible. Otherwise, the dried samples should be stored in a desiccator for a short time under vacuum.

10. Sputter coating and FESEM imaging:
  a. Transfer the silicon chip onto aluminum stab covered with a carbon adhesive tab, and then transfer it to a sputter-coating apparatus.
  b. Coat the specimens with 10-nanometre thick gold for FESEM imaging.
    ***Note:*** About 3-nm thick chromium or 2-nm thick iridium coat is reported to be more suitable for other nuclei specimens.^5^^,^^6^ In zebrafish early embryonic nuclei, we tested 3-nm thick chromium coat, but the conductivity of coated specimens was relatively poor. Therefore, we chose to use a 10-nm thick gold coat, though it slightly reduced resolution.
  c. Image NPCs on the nuclear surface under a FESEM instrument (e.g., FEI Helios NanoLab G3 UC) after professional training or with assistance from an instrumental technician. Troubleshooting 5.

### Isolation of pure nuclei (optional)

**Timing: 3 h**

This step is optionally performed for additional purification of nuclei after hypotonic treatment (step 5). The usage of a dense sucrose solution followed by high-speed centrifugation would help separate nuclei from mitochondria and residual plasma membrane in the crude pellet fraction based on their densities. We purified intact nuclei from zebrafish 32-cell and 512-cell stage embryos with a successful rate of 1.5% (number of isolated intact nuclei/total number of cells) and successfully used them for mass spectrometry analysis.^1^

11. Discard the supernatant with liquid residual as little as possible.
12. Gently resuspend the pellet in hypertonic sucrose buffer:
  a. Add 500 μL hypertonic sucrose buffer to the pellet.
  b. Gently pipette the pellet using a 200-μL tip for suspension.
  c. Transfer the suspension to the cylinder of a Dounce homogenizer.

***Note:*** Using high concentration of sucrose helps prevent diffusion of proteins into the homogenizing medium.

13. Homogenize the suspension in a Dounce homogenizer with the “loose” or named “A” pestle for 8–10 passes, up and down the cylinder, on ice.

***Note:*** Zebrafish early embryonic nuclei are slightly larger than those of somatic cells. Therefore, the “loose” but not the “tight” pestle should be used in order to maintain intactness of nuclei.

***Note:*** For zebrafish early embryos, up to 10 passes through the Dounce homogenizer are sufficient and keep most nuclear membrane intact. For other cell types, the number of strokes should be optimized to preserve both sufficiency and the nuclear membrane.

14. Transfer the homogenate to a 1.5 mL polypropylene ultracentrifuge tube (Beckman 357448) using a 200-μL pipette.

***Note:*** Avoid using conventional 1.5-mL EP tubes, which are incapable of withstanding ultracentrifugation.

15. Centrifuge the homogenate at 80,000 × *g* for 80 min at 4°C.

***Note:*** This step aims to separate nuclei from mitochondria, membranes, and yolk remnants based on density.

16. Aspirate the supernatant sucrose buffer as far as possible, especially the residuals on the tube wall. Take care not to disturb the nuclei pellet at the bottom.

***Note:*** The number of nuclei isolated from zebrafish early embryos is very limited, so the pellet is typically invisible to the naked eye. The nuclei pellet is expected to sediment toward the bottom of the EP tube along the direction of centrifugal force.

17. Resuspend the pellet in 205 μL PBS by gently pipetting using a 200-μL tip. Transfer the resuspension to a new 1.5-mL EP tube.

***Note:*** PBS without Triton X-100 was used to preserve the nuclear envelope, which contained embedded proteins of interest like nucleoporins.

***Optional:*** To remove the nuclear envelope, use PBS + 0.5% Triton X-100. However, the addition of Triton X-100 or other detergents would increase the tendency of nuclei aggregation, which is hard to be disassociated into individual nucleus.

18. Nuclei collection.
  a. Centrifuge 200 μL suspension at 800 × *g* for 10 min at 4°C to pellet the nuclei.
  b. Discard the supernatant. Aspirate PBS absolutely, but don’t touch the potential nuclei pellet.
  c. Snap freeze in liquid nitrogen, and store at −80°C for up to 1 month.

***Note:*** Leave 5 μL nuclei suspension for subsequent number counting and quality check.

**Figure 4:**
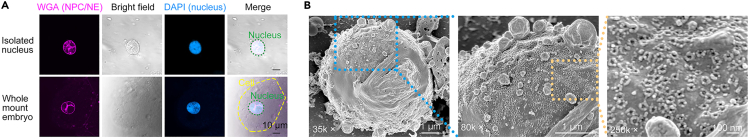
Representative images of isolated nucleus and NPCs embedded in NE (A) Representative confocal images for quality check of isolated nuclei from 512-cell embryos. The quality of an intact isolated nucleus is determined by WGA and DAPI staining as well as bright field observation. WGA, FG-Nups for labeling NE integrity; DAPI, DNA for labeling nucleus; bright field, reflecting well isolated nucleus devoid of cytoplasm (upper) or nucleus in the whole cell (bottom). Green dotted circle, nucleus; yellow outline, boundary of a cell. Scale bar, 10 μm. (B) Representative images of NPCs embedded in NE from 512-cell embryos by FESEM. Magnification values are displayed in the lower-left corner of each image. Enlarged regions are denoted by dotted boxes. Scale bar, 1 μm (left and middle) and 100 nm (right).
  d. Count the number of nuclei with a hemocytometer. Estimate the total number of collected nuclei based on the whole volume.
20. Record the location of storage and the number of nuclei for each tube.

***Note:*** Different tubes containing the same sample could be pooled to obtain sufficient nuclei for subsequent mass spectrometry.

19. Quality check and number counting of isolated nuclei.
  a. Add 5 μL PBS to the remaining 5 μL resuspension to expand the volume.
  b. Stain the isolated nuclei with WGA AF647 (final concentration of 5 μg/mL) and DAPI (final concentration of 1 μg/mL) for 10 min at room temperature.
  c. Transfer about 2 μL nuclei suspension into a confocal dish and take photos under a confocal microscope for quality check (Figure 4A).
    ***Note:*** WGA labels FG-Nups, which reflects the integrity of NE; bright field images show the successful exposure of nuclei; and DAPI labels DNA to indicate the nucleus.

21. Perform mass spectrometry analysis.

***Note:*** Obtaining nuclei from zebrafish early embryos was largely limited by cell number and isolation efficiency. We used an optimized label-free mass spectrometry method with about 12500 nuclei per sample.

## Expected outcomes

We expect to obtain images of exposed nuclei located on the silicon chip, and NPCs on the cytoplasmic side of the NE can be seen (Figure 4B). In our experience, the number of nuclei released from zebrafish early embryos is typically low due to limited number of interphase cells. Furthermore, the morphology of NPCs on NE is extremely heterogeneous.

## Quantification and statistical analysis

### Analysis of different NPC type ratios

This step describes the statistical analysis of different NPC types on the NE using the imageJ Fiji plugin “Cell Counter”.^8^ NPC types are classified according to previous reports.^10^^,^^11^ Dimples, stable pores, rosette, star rings, and thin rings are regarded as structural intermediates of NPCs, which may be generated during assembly or disassembly process. Ratios of different NPC intermediates on the NE would reflect NPC heterogeneity and comprehensive maturity.

1. Open the FESEM image with imageJ Fiji software (Figure 5A).

**Figure 5:**
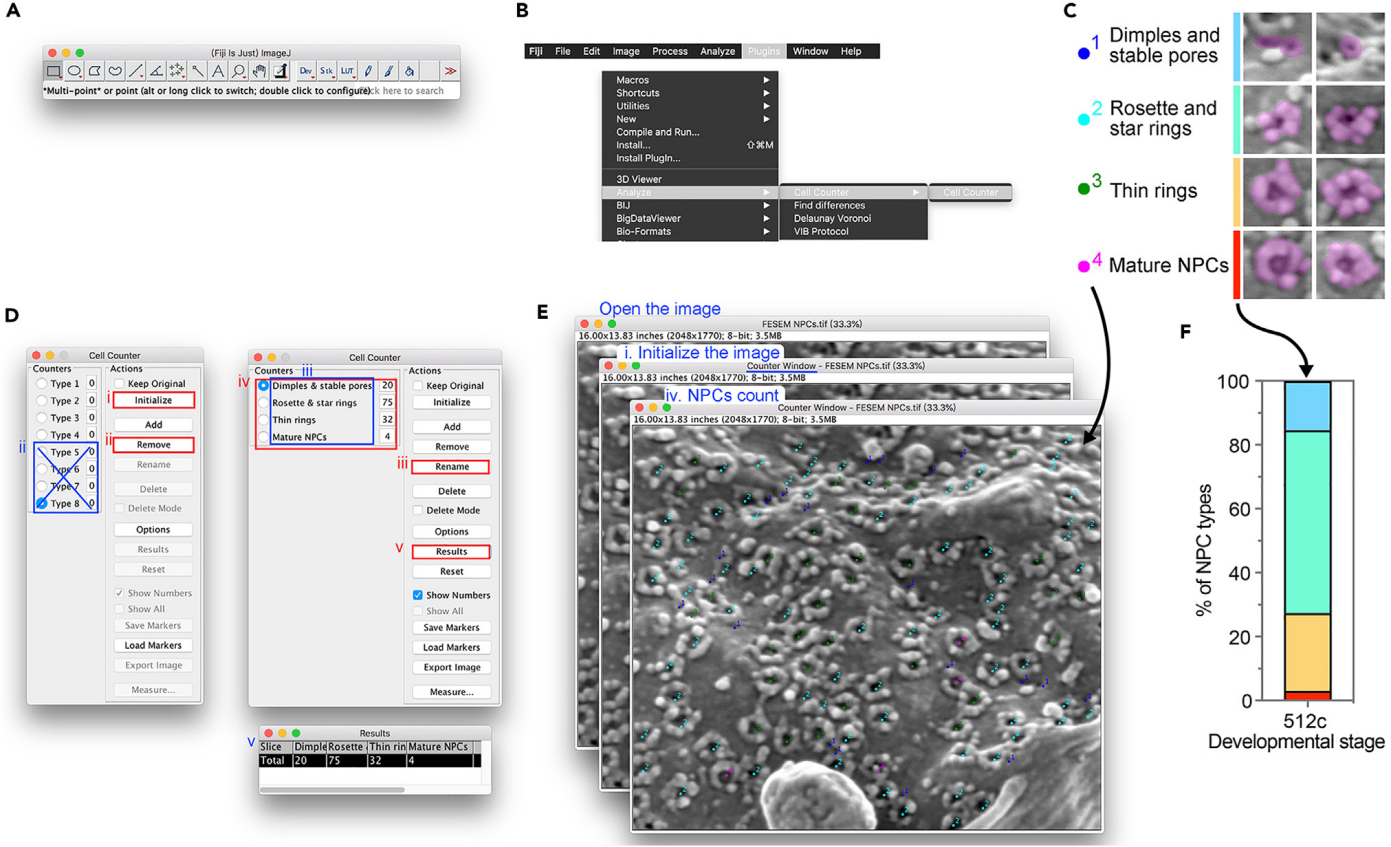
Data analysis of different NPC types (A and B) Data analysis is performed in imageJ Fiji software with a plugin “Cell Counter” (Plugins/Analyze/Cell Counter/Cell Counter). (C) Representative images of different NPC types. NPCs are masked by transparent magenta. The colors of the dots and the bars correspond to those in (E) and (F), respectively. (D and E) Count the number of different NPC types. Operating steps are labeled in red, and relevant results are labeled in blue. The example image is from 512-cell embryos, the same as that in Figure 4. (F) Fraction of different NPC types in the example image.
2. Use “Cell Counter” plugin (Plugins/Analyze/Cell Counter/Cell Counter) to record different types of NPCs on the NE (Figure 5B).
3. Classify and count different NPC types.
  a. Define different NPC types as dimples and stable pores, rosette and star rings, thin rings, and mature NPCs, which are characterized by size, cytoplasmic ring and central channel (Figure 5C).
  b. Count the number of NPCs of different types (Figures 5D and 5E).
    i. Run “Initialize” for selected image initialization.
    ii. Select unnecessary types and run “Remove” to delete extra types.
    iii. Select a specific type and run “Rename” to rename the type for convenient record.
    iv. Count different NPC types by left clicking on the image where a specific NPC type is located after selection of the corresponding type.
    v. Run “Results” and export the data.
4. Perform statistical analysis in Prism software (Figure 5F).
  a. Paste the above result into Prism “Data table”.
  b. Perform fractional analysis (Analyze/Fraction of Total).
  c. Plot the fraction result in stacked bars.

Fractions of different NPC types in the nucleus at a particular developmental stage were therefore statistically determined and presented. Comparative results from different developmental stages or different genetic backgrounds would be obtained in this way, which would reflect differences in NPC states and complexity.

## Limitations

The hypotonic treatment-based method described here is optimized for zebrafish early embryos. It generally also works very well in most culture cells or zebrafish embryonic cells after MBT. However, we have not tested whether this protocol is applicable to early embryonic cells from other species such as *Drosophila*, *Xenopus* or mouse. Additionally, the number of exposed nuclei spreading on the silicon chip is usually low due to at least three reasons: 1) the number of cells in early embryos is limited; 2) only a small fraction of cells stay in interphase due to fast and synchronous cleavage (approximately 15 min per cycle) before MBT; and 3) a low nucleus-cytoplasm ratio before MBT indicates that a large proportion of cytoplasm would occupy the territory of the nucleus on the surface of silicon chip. In addition, NPCs in isolated nuclei are reported to be constricted compared to that in native cellular environment.^12^^,^^13^^,^^14^ Furthermore, this protocol provides an overview of the cytoplasmic side of NPCs, whereas the nuclear side of NPCs remains unknown.

## Troubleshooting

### Problem 1

The number of embryos at a specific stage may not be sufficient (section ‘‘Fertilized eggs collection at 15-min interval’’, step 2).

### Potential solution

A large number of embryos at a specific early stage are required for nuclei exposure, especially for isolation of pure nuclei. However, the rapid and synchronous cleavage cell cycle before MBT limits the acquisition of sufficient embryos at the same developmental stage. Some preparations could be done as follows (Figures 1A–1C):

• Prepare more breeding fish;
• Release all breeding fish at the same time point so that spawning time points would be very close;
• Collect fertilized eggs at an interval of 15 min;
• Repeat egg collection for 3–4 times.

### Problem 2

It is difficult to perform experiments on embryos of different stages on the same day (section ‘‘Fertilized eggs collection at 15-min interval’’, step 2).

### Potential solution

If fertilized eggs are collected at the same time, embryos at different stages have to be harvested at different time points, which disallows analysis of all these embryos side by side. A solution would be to stagger the spawning time and perform nuclei exposure experiments at the same time. In other words, determine the desired developmental stages for nuclei collection and the breeding schedule, then collect embryos of different desired stages at the same time after confirming embryonic morphology (Figure 1D).

### Problem 3

The intact nuclear envelope is possibly ruptured, especially from embryos before MBT (section “Nuclei exposure of zebrafish early embryos”, step 4).

### Potential solution

The NE of zebrafish early embryos is more fragile. An initial short exposure to low concentration of PFA would greatly protect the intact NE and increase the nuclear isolation efficiency. Furthermore, pay attention to gentle operation throughout the whole process.

### Problem 4

Air drying causes distortion and damage to aqueous biological samples (section “Nuclei collection and sample preparation for FESEM”, step 7).

### Potential solution

Keep samples immersed in liquid and protected from drying all the time before the CPD process. For solution exchange, we transfer the silicon ship between two wells with pre-filled solution. Optionally, use two pipettes for solution exchange, one for removing old solution, and another for introducing new solution simultaneously. The tip of the pipette should not touch the silicon chip.

### Problem 5

The exposed nuclei on the silicon chip are usually in low number, especially for embryos at very early stages (section “Nuclei collection and sample preparation for FESEM”, step 10).

### Potential solution

Performing effective deyolking and doing filtration through a 40 μm cell strainer would reduce remnants, thereby leaving more areas for nuclei attachment on the silicon chip surface. Collection of more embryos is one potential way, but too many embryos would introduce more remnants. Furthermore, increasing nuclei exposure efficiency is also useful, including short exposure to low concentration of PFA, gentle manipulation, and the addition of protease inhibitors.

## Resource availability

### Lead contact

Further information and requests for resources and reagents should be directed to and will be fulfilled by the lead contact, Anming Meng (mengam@mail.tsinghua.edu.cn).

### Materials availability

This study did not generate new unique reagents.

## Data Availability

This study did not generate new datasets or codes.
